# Body size and temperature effects on standard metabolic rate for determining metabolic scope for activity of the polychaete *Hediste (Nereis) diversicolor*

**DOI:** 10.7717/peerj.5675

**Published:** 2018-10-29

**Authors:** Helena Lopes Galasso, Marion Richard, Sébastien Lefebvre, Catherine Aliaume, Myriam D. Callier

**Affiliations:** 1 UMR MARBEC UM CNRS Ifremer IRD, Palavas les Flots, France; 2 CAPES Foundation, Ministry of Education of Brazil, Brasília, Brazil; 3 UMR MARBEC UM CNRS Ifremer IRD, Montpellier, France; 4 UMR MARBEC UM CNRS Ifremer IRD, Sète, France; 5 Université de Lille, CNRS, ULCO UMR8187 LOG (Laboratoire d’Océanologie et Géosciences), Wimereux, France; 6 Ifremer, Laboratoire Ressources Halieutiques, Boulogne sur mer, France

**Keywords:** Annelida, Deposit-feeder, Oxygen consumption, Allometric coefficient, Arrhenius temperature, Aerobic scope, Growth

## Abstract

Considering the ecological importance and potential value of *Hediste diversicolor*, a better understanding of its metabolic rate and potential growth rates is required. The aims of this study are: (i) to describe key biometric relationships; (ii) to test the effects of temperature and body size on standard metabolic rate (as measure by oxygen consumption) to determine critical parameters, namely Arrhenius temperature (*T_A_*), allometric coefficient (*b*) and reaction rate; and (iii) to determine the metabolic scope for activity (MSA) of *H. diversicolor* for further comparison with published specific growth rates. Individuals were collected in a Mediterranean lagoon (France). After 10 days of acclimatization, 7 days at a fixed temperature and 24 h of fasting, resting oxygen consumption rates *(VO_2_*) were individually measured in the dark at four different temperatures (11, 17, 22 and 27 °C) in worms weighing from 4 to 94 mgDW (*n* = 27 per temperature). Results showed that DW and L3 were the most accurate measurements of weight and length, respectively, among all the metrics tested. Conversion of WW (mg), DW (mg) and L3 (mm) were quantified with the following equations: DW = 0.15 × WW, L3 = 0.025 × TL(mm) + 1.44 and DW = 0.8 × L3^3.68^. Using an equation based on temperature and allometric effects, the allometric coefficient (*b*) was estimated at 0.8 for DW and at 2.83 for L3. The reaction rate (*VO_2_*) equaled to 12.33 µmol gDW^−1^ h^−1^ and 0.05 µmol mm L3^−1^ h^−1^ at the reference temperature (20 °C, 293.15 K). Arrhenius temperature (*T_A_*) was 5,707 and 5,664 K (for DW and L3, respectively). Metabolic scope for activity ranged from 120.1 to 627.6 J gDW^−1^ d^−1^. Predicted maximum growth rate increased with temperature, with expected values of 7–10% in the range of 15–20 °C. MSA was then used to evaluate specific growth rates (SGR) in several experiments. This paper may be used as a reference and could have interesting applications in the fields of aquaculture, ecology and biogeochemical processes.

## Introduction

Polychaetes play important roles in marine and brackish ecosystem functioning both as predator and prey in food webs (Kristensen, 1983a) or in biogeochemical cycles as detritivores and bioturbators (Mermillod-blondin, François-Carcaillet & Rosenberg, 2005; e.g., Hedman et al., 2011). Among these polychaete species, *Hediste (Nereis) diversicolor* has been the most studied in terms of ecology and ecotoxicology (Kristensen, 1984; Scaps, 2002; Mermillod-blondin, François-Carcaillet & Rosenberg, 2005; Mouneyrac et al., 2009). Conducive to farming, *H. diversicolor* is grown as animal feed and bait for recreational fishing (Fidalgo e Costa, Narciso & Cancela da Fonseca, 2000) or in order to reduce organic waste in integrated multi-trophic aquaculture (IMTA) (Batista et al., 2003; Bischoff, Fink & Waller, 2009).

Considering the ecological interest and potential economic value of *H. diversicolor,* predicting its metabolism and growth in a wide range of environmental or culture conditions is critical. Metabolism is often proxied by oxygen consumption rates and some figures are available in the literature for *H. diversicolor* (Kristensen, 1981; Fritzsche & Von Oertzen, 1995) but are highly disparate. Fritzsche & Von Oertzen (1995) observed the lowest oxygen rates for *H. diversicolor* and Shumway (1979), Kristensen (1983a) and Nielsen et al. (1995) the highest. Differences in experimental conditions and the physiological state of the individuals may explain these large discrepancies. Oxygen measurements were carried out on starved worms in washed, burnt sediment by Fritzsche & Von Oertzen (1995). Other authors also used unfed worms but they were being kept in artificial plexiglass or polyethylene tubes (Kristensen, 1983a; Nielsen et al., 1995) and light conditions are not specified. Light has been shown to influence polychaete metabolism with lower means observed at night (Sander, 1973). Ventilation amplitude and duration of two species of *Nereis* were lower in natural sediment than in artificial tubes (Kristensen, 1983b). The metabolic rate can vary between two fundamental physiological rates: the standard metabolic rate (SMR) defined as the minimal maintenance metabolic rate below which physiological function is impaired, and the active metabolic rate (AMR) which is the maximum aerobic metabolic rate, SMR and AMR being measured at a given temperature and for a given individual weight (Norin & Malte, 2011). Accordingly, metabolic rates of polychaetes may be four- to six-fold higher during activity than at rest, e.g., *Diopatra cuprea* (Mangum & Sassaman, 1969) or *H. diversicolor* (Kristensen, 1981), respectively. Most published values on *H. diversicolor* probably correspond to AMR, incorporating energy cost from ventilation, behavior, digestion and light-related stress; in contrast, SMR has received little attention. The difference between AMR and SMR gives the metabolic scope for activity (MSA also named aerobic scope for activity or absolute aerobic scope, see Norin & Malte, 2011) which is the potential for any energy-requiring work, i.e., behavior, growth, physiological regulation, fighting disease and other stresses (Claireaux & Lefrançois, 2007). Metabolic scope for activity is strongly correlated to maximum growth rates (see Lefebvre et al., 2001 in Claireaux & Lefrançois, 2007) and this parameter is a good indicator of fitness and animal welfare in natural and aquaculture conditions, with relevance to growth performance, habitat selection, energy acquisition, energy allocation and stress.

Beyond measuring SMR and AMR, making a robust MSA estimate requires the establishment of a temperature law for the given species and a coherent allometric coefficient. As for all ectothermic species, temperature affects *H. diversicolor* metabolism and behavior, i.e., growth rate (Neuhoff, 1979), depth of gallery construction (Esselink & Zwarts, 1989) and ventilation activity (Kristensen, 1983a). Van’t Hoff (*Q*_10_ values) and Arrhenius laws (see Kooijman, 2010) are useful when converting temperature-dependent physiological rates. However, no effects of temperature and concomitant allometric laws have been published on this species. Metabolic rate is proportional to body mass with an allometric coefficient *b,* ranging from $ \frac{2}{3} $ to $ \frac{3}{4} $ (Glazier, 2005; Makarieva et al., 2008). The *b* is typically higher in younger individuals (up to 1) than older ones (down to $ \frac{2}{3} $; Riisgård, 1998). For *H. diversicolor*, *b* was estimated at 1.20 for individuals weighing between 30 and 90 mg dry weight (DW) (Nielsen et al., 1995) and at 0.69 for individuals weighing between 80 and 220 mgDW (Shumway, 1979). Part of the variation in *b* values within species can be also attributed to other non-uniform experimental conditions (temperature, state of nutrition, salinity; see a review in Glazier, 2005). Thus, there is a real need of data that simultaneously describe *H. diversicolor* size-weight relationships and metabolic rates for a large range of body sizes and temperatures in the same experimental conditions. Finally, a neglected complication for polychaetes is that their biometrics are often reported using different metrics of weight (dry or wet weight) or length (total length, number of segment, L3: length of three first segments) (Kristensen, 1984; Gillet, 1990; Durou, Mouneyrac & Amiard-Triquet, 2008) but no paper explains the relationships between all of these metrics to make it easy to compare metabolic rates standardized by weight (Shumway, 1979; Kristensen, 1983a; Fritzsche & Von Oertzen, 1995; Nielsen et al., 1995) or length (Dales, 1950; Ozoh & Jones, 1990).

In this study, we aimed to evaluate the simultaneous effects of temperature and body size on the metabolic scope for activity of *H. diversicolor.* Specific aims were (i) to evaluate the effect of body size and temperature on standard metabolic rate (SMR) by measuring oxygen consumption by unfed worms kept in the dark with no special tubes, to favor resting time as in Kristensen (1981), (ii) to quantify relationships between *H. diversicolor* body weight and length metrics*,* (iii) to determine the Arrhenius and Van’t Hoff laws for temperature effects, and (iv) to estimate metabolic scope for activity for a given weight at different temperatures by comparison with active metabolic rate (AMR) values in literature, and predict potential maximum growth rates. This study will provide useful data on *H. diversicolor* biometrics, metabolic rate and scope for activity, and ultimately be an indicator for understanding growth rates in aquaculture or natural conditions.

## Materials & Methods

### Specimen sampling

Specimens of *H. diversicolor* were collected in November 2015 from the Arnel lagoon near Villeneuve-les-Maguelone, France (43°31′11.8″N 3°53′03.5″E). The salinity and temperature of the lagoon water were 18 psu and 18 °C, respectively. Sediment was collected using a core from a depth of 30 to 40 cm and sieved *in situ* with a 500 µm mesh. Individuals were identified, transferred into plastic containers with seawater and then transported to the Ifremer experimental station (Palavas-les-Flots). In the lab, the worms were kept at a density of 6,250 ind m^−2^ in small, continuously aerated 5-L aquariums filled with filtered seawater (salinity 18 psu, temperature 18 °C). The polychaetes were acclimatized for 10 days and fed *ad libitum* with seabass *Dicentrarchus labrax* faeces (pretreated by centrifugation for 5 min at 5,000 rpm). The seabass faeces consisted of 63% organic matter, 6% lipids, 19% protein content (deduced from 3% total organic nitrogen), 31% total organic carbon and 2% total phosphorous (% of dry weight) (Galasso et al., 2017). To reduce stress and conflict between worms, cylindrical plastic tubes (∅ = 4 mm of varying lengths) were placed in the aquariums to serve as individual shelters.

### Biometric measurements and relationships

All biometric measurements were performed on individuals after a fasting period of 24 h to empty their digestive tracts. Worms were carefully taken using a dip net, cleaned and dried on paper towels, before being weighed on a precision balance to obtain the individual wet weight (WW). Before metabolic measurements, individuals were grouped by WW into three size classes: 0–0.15 g, 0.15–0.30 g and 0.30–0.60 g, then put back into the same culture conditions as previously described. Each individual was photographed to determine L3 (combined length of three first segments: i.e., the prostomium, peristomium and first chaetiger) using a Canon EOS 1100D digital camera and the images were treated with ImageJ software (public domain digital analysis software ImageJ 1.42q; National Institutes of Health, Bethesda, MD, USA). At the end of the incubation period for the oxygen consumption measurements, WW and L3 were once again measured, and then the polychaetes were dried at 50 °C for 24 h to obtain individual dry weights (DW). Total length (TL) and number of segments (NSEG) were also determined by image treatment. Biometric measurements were correlated with each other to determine relationships between (a) dry and wet weight, (b) several proxies of length and (c) weight and length.

### Experimental design

The effect of body size (continuous variable) and temperature (fixed variable, 4 levels) on individual metabolic rate was measured. As specified in ‘Biometric Measurements and Relationships’, three representative body size classes were defined to classify the spectrum of individuals. The temperatures tested were 11, 17, 22 and 27 °C using four 30-L aquariums (35 × 60 × 30 cm). These temperatures were chosen based on temperature variation in *H. diversicolor* habitat and aquaculture systems. The water in two aquariums was heated to 22 and 27 °C using immersion heaters, 17 °C corresponded to constant room temperature and in the last aquarium, the water was cooled to 11 °C using a refrigeration unit. The second acclimatization period for the polychaetes was to adapt them to the different temperatures and lasted 7 days. During this time, the worms were also fed *ad libitum* with seabass faeces and water salinity was maintained at 18 psu. Worms were acclimatized into 1L-microcosms at 3 ind L^−1^ with their respective plastic tubes for shelter. Air diffusers were placed in each microcosm to aerate the water. Pumps were also introduced to mix the water in the aquariums to ensure the temperature remained uniform in each microcosm. Worm body size was randomly attributed to microcosms. Each body size class was replicated three times. A total of 108 worms was used in this experiment (four temperatures, three body class sizes, three microcosms, three worms). No significant difference was observed according to temperature for DW (*p* = 0.4), WW (*p* = 0.5), L3 (*p* = 0.7), TL (*p* = 0.7), NSEG (*p* = 0.2) or DW:L3 ratio (*p* = 0.5) after the acclimatization period, i.e., during the oxygen consumption measurement phase.

### Individual standard metabolic rates

Standard metabolic rates (SMR) were measured individually using 36 chambers plus three control chambers containing water without polychaetes at each temperature. Another four aquariums were used as incubators (11, 17, 22 and 27 °C) for chambers and were filled with filtered seawater (salinity 18 psu) previously saturated with air. They were kept in the dark to favor resting time, avoid worm stress and inhibit oxygen generation by autotrophic organisms. All 144 measurements (108 worms + 36 controls) were made over three days. Worms were fasted for 24 h before the experiment.

SMR was proxied by oxygen consumption rate and measurements were made using chambers (125-mL glass bottles *H* = 102 mm, Ø= 51 mm) with rubber-sealed polypropylene caps (Fisher Brand 2911475) fitted with optodes so a fiber optic oxygen transmitter (Fibox 4 trace, PreSens—Precision Sensing GmbH, Regensburg, Germany) could obtain a phase measurement which was later converted to oxygen concentration (Richard et al., 2017). The optodes were previously calibrated with an oxygen-saturated solution and an oxygen-free solution (containing Mn(OH)_2_). An initial measurement (*t*_0_) was obtained, then the chambers were placed in the incubation aquariums. Incubation times (chosen to prevent oxygen saturation dropping below 80% to avoid physiological stress) varied from 4 h (for the aquariums at 22 and 27 °C) to 5 h (for those at 11 and 17 °C). At the end of the incubation period (*t*_*f*_), the chambers were moved up and down to mix the water before measurement of the final oxygen concentration.

Oxygen consumption rates were then calculated using the following equation:

\begin{eqnarray*}V{O}_{2}= \frac{ \left[ \left( {{O}_{2}}_{ \left( {t}_{f} \right) }-{{O}_{2}}_{ \left( {t}_{0} \right) } \right) - \left( c{{O}_{2}}_{ \left( {t}_{f} \right) }-c{{O}_{2}}_{ \left( {t}_{0} \right) } \right) \right] \times Vol}{ \left( {t}_{f}-{t}_{0} \right) } \end{eqnarray*}

where *VO*_2_ is oxygen consumption rate (µmol h^−1^), ${{O}_{2}}_{ \left( {t}_{f} \right) }$ is oxygen concentration at *t*_*f*_ (µmol L^−1^), ${{O}_{2}}_{ \left( {t}_{0} \right) }$ is oxygen consumption at *t*_0_ (µmol L^−1^), $ \left[ {{cO}_{2}}_{ \left( {t}_{f} \right) }-{{cO}_{2}}_{ \left( {t}_{0} \right) } \right] $ is the difference between oxygen concentration measured in control chamber at *t*_*f*_ and *t*_0_ (µmol L^−1^), *Vol* is the volume of the individual incubation chambers (L), *t*_0_ is the initial incubation time and *t*_*f*_ is the final incubation time (h).

### Effect of body size and temperature on standard metabolic rate

Metabolism is proportional to a constant power of the body weight or length as described by the allometric equation *y* = *ax*^*b*^ where *y* is the metabolic rate (measured as oxygen consumption), *x* is the body weight or length, *b* is the allometric coefficient and *a* corresponds to the metabolic rate of an animal per unit of weight (*VO*_2_ or $\dot {k}$ also called reaction rate). Accordingly, reaction rate (oxygen consumption) curves were constructed using the general model below accounting for the effect of temperature (Arrhenius relationship) and body size scaling:

\begin{eqnarray*}\dot {k}(T,X)={\dot {k}}_{1}\exp \nolimits \left( \frac{{T}_{A}}{{T}_{1}} - \frac{{T}_{A}}{T} \right) {X}^{b} \end{eqnarray*}

where *T* is the absolute temperature (in Kelvin: °C + 273.15 °K), *T*_1_ is a chosen reference temperature (here 20 °C, 293.15 °K), *T*_*A*_ is the Arrhenius temperature, $\dot {k}$ is the reaction rate and ${\dot {k}}_{1}$ its value at the reference temperature *T*_1_, *X* is a body size dimension (WW, DW, L3, TL or NSEG), and *b* is the scaling exponent of the allometric effect.

Curve fitting was achieved using the downhill simplex method of the Nelder–Mead model and standard deviations were estimated using an asymptotic method with MATLAB R2010b. All fits were tested by analysis of variance (*p* < 0.001) with residuals being tested for normality and homogeneity of variance as well as parameter significance testing using Student’s *t*-test (*p* < 0.05).

The Van’t Hoff coefficient (*Q*_10_) is the factor that should be applied to reaction rates for every 10 °C increase (Kooijman, 2010) and it was determined according to:

\begin{eqnarray*}\dot {k}(T)={\dot {k}}_{1}{Q}_{10}^{ \frac{(T-{T}_{1})}{10} } \end{eqnarray*}

where $\dot {k}$ is reaction rate, *T* the absolute temperature (in Kelvin) and *T*_1_ a chosen temperature. The relationship between *Q*_10_ and *T*_*A*_ is the following (Kooijman, 2010):

\begin{eqnarray*}{Q}_{10}=\mathrm{exp} \left[ \frac{10{T}_{A}}{T \left( T+10 \right) } \right] . \end{eqnarray*}

### Comparison with literature data and determination of active metabolic rate

Measured SMR values were compared to literature data. Original data were extracted from publications using the freeware PlotReader (Bruggeman, 2010). Published *H. diversicolor* oxygen consumption rates were converted to µmol h^−1^ using 1 mol O_2_ = 22.4 L = 32 g = 437.67 kJ. Oxygen consumption rates were first standardized at 20 °C using Arrhenius law (Kooijman, 2010), reaction rate and the previously determined allometric coefficient. Then, relationships between oxygen consumption rate at 20 °C (${\dot {k}}_{1}$ in µmol h^−1^) and worm weight (DW in g) were established. When necessary, DW was converted into WW using the relationship established in this study. Ash free dry weight (AFDW) was converted into DW using *DW* = *AFDW* × 1.28 (Wetzel, Leuchs & Koop, 2005).

Oxygen consumption for a standard individual of 1 gDW ($\dot {k}$) was determined from observations and literature data using the equation: $\dot {k}=(Ws/We)^{b}Ke$ where $\dot {k}$ is the standard oxygen consumption (µmol gDW^−1^ h^−1^), *Ws* is the standard weight (1 gDW), *We* is the observed weight, *Ke* is the observed oxygen rate and *b* the allometric coefficient determined earlier. Mean (±SE) $\dot {k}$ also called *VO*_2_ were next reported to observed temperature. Data from this study were adjusted to the temperature range of Kristensen (1983a) (4.7, 10.98, 15.3, 19.5 and 29.5 °C). Higher oxygen consumption rates reported in the literature were assumed to represent the active metabolic rate (AMR).

### Metabolic scope for activity and maximum specific growth rates

The metabolic scope for activity (MSA) reflects the capacity of the respiratory system to provide energy for all activities beyond standard metabolism, e.g., growth, reproduction and physical activity. MSA was determined by subtracting standard metabolic rate (SMR corresponding to our data measured for fasting, resting worms in the dark) from active metabolic rate (AMR corresponding to the highest oxygen rate reported in the literature) according to temperature for a given individual weight. Data were converted into DW (g) and calculated for each temperature used for calculation of AMR (see above in ‘Comparison with literature data and determination of active metabolic rate’).

Maximum specific growth rates (*μ*_max_ d^−1^) were determined by comparing MSA with energy cost per unit of growth (*n* = J gDW^−1^). Energy cost per unit of growth (*n*) is defined as slope of metabolic rate (J gDW^−1^ d^−1^) against  *μW*^(1−*b*)^ where  *μ* corresponds to growth rate (% d^−1^), *W* to weight (DW in g) and *b* to the allometric coefficient (see Kiørboe, Munk & Richardson, 1987 in Nielsen et al., 1995). Nielsen et al. (1995) calculated the energy growth cost per unit of growth of *H. diversicolor* as 5085.6 J gDW^−1^, excluding data related to negative growth rates and considering *b*, the allometric coefficient determined in this study. Published growth rates were then compared to predicted  *μ*_max_.

## Results

### Biometric measurements

**Figure 1:**
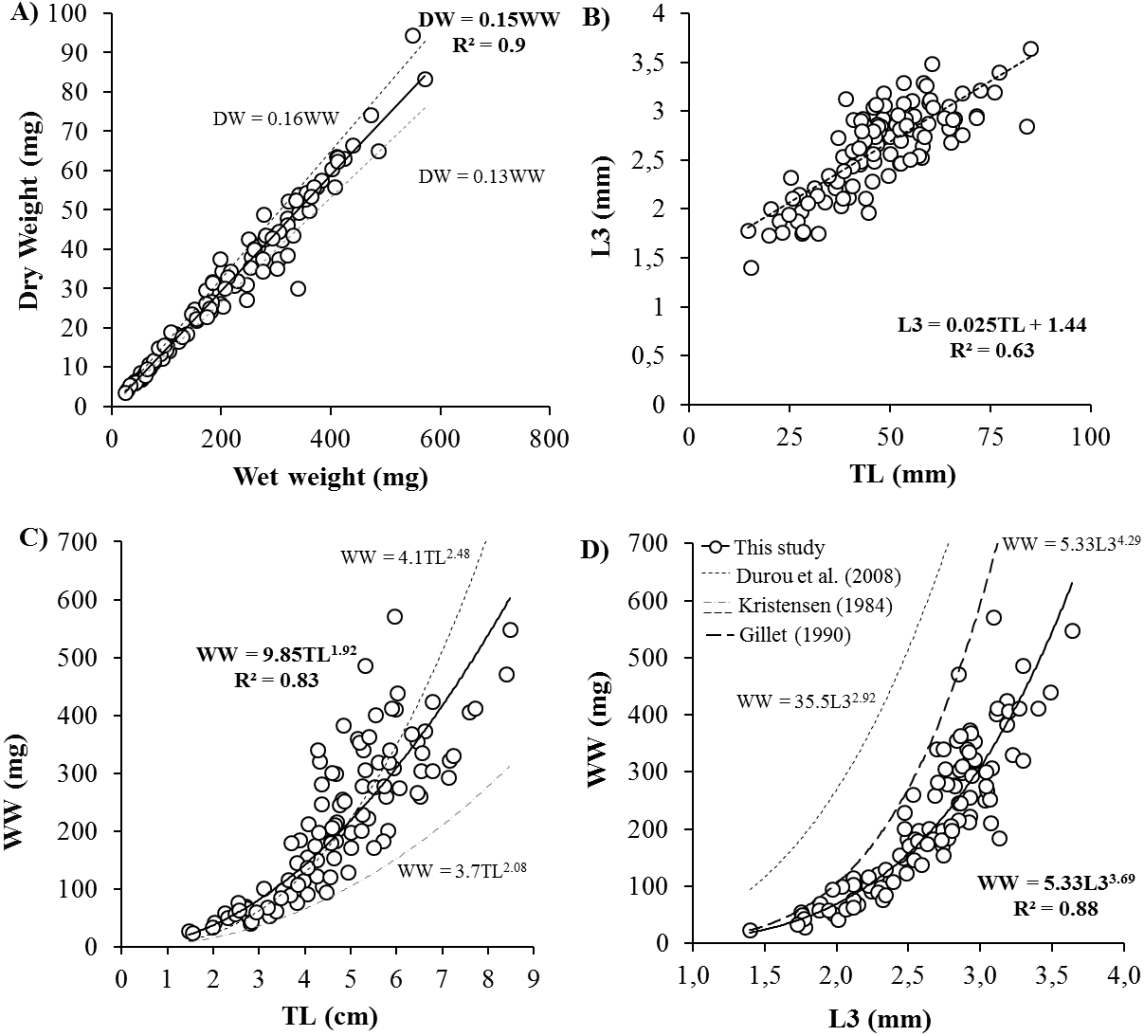
Biometric measurements relationships for *Hediste diversicolor*. Key relationships for *Hediste diversicolor* between body weight and length: (A) dry vs. wet weight (DW vs. WW), (B) length of three first segments vs. total length (L3 vs. TL), (C) WW vs. TL and (D) WW vs. L3. *R*^2^ is the determination coefficient in (A, B) as linear relation *y* = *ax* and in (C, D) as power law *y* = *ax*^*b*^ represented by continuous lines. Size-weight relationships described by Durou, Mouneyrac & Amiard-Triquet (2008), Kristensen (1984) and Gillet (1990) for *H. diversicolor*.

Body size ranged from 20 to 570 mg WW, 4 to 94 mgDW, 1.4 to 3.6 mm L3, 14.6 to 84.8 mmTL and 23 to 84 NSEG. Key biometric relationships showed significant linear correlations between DW and WW, TL and L3 (Fig. 1). Relation between total length (mm) and number of segments was TL = 0.85 × NSEG (*R*^2^ = 0.55). After logarithmic transformation, DW and WW significantly correlated with length measurements with higher determination coefficients for L3 (DW (mg) = 0.8 × L3 (mm)^3.68^, *R*^2^ = 0.88) than TL (DW (mg) = 1.5 × TL (cm)^1.90^, *R*^2^ = 0.81) and NSEG (DW (mg) = 1.2 × 10^−5^ NSEG^1.93^, *R*^2^ = 0.45).

### Effect of body size and temperature on standard metabolic rate (SMR)

**Figure 2:**
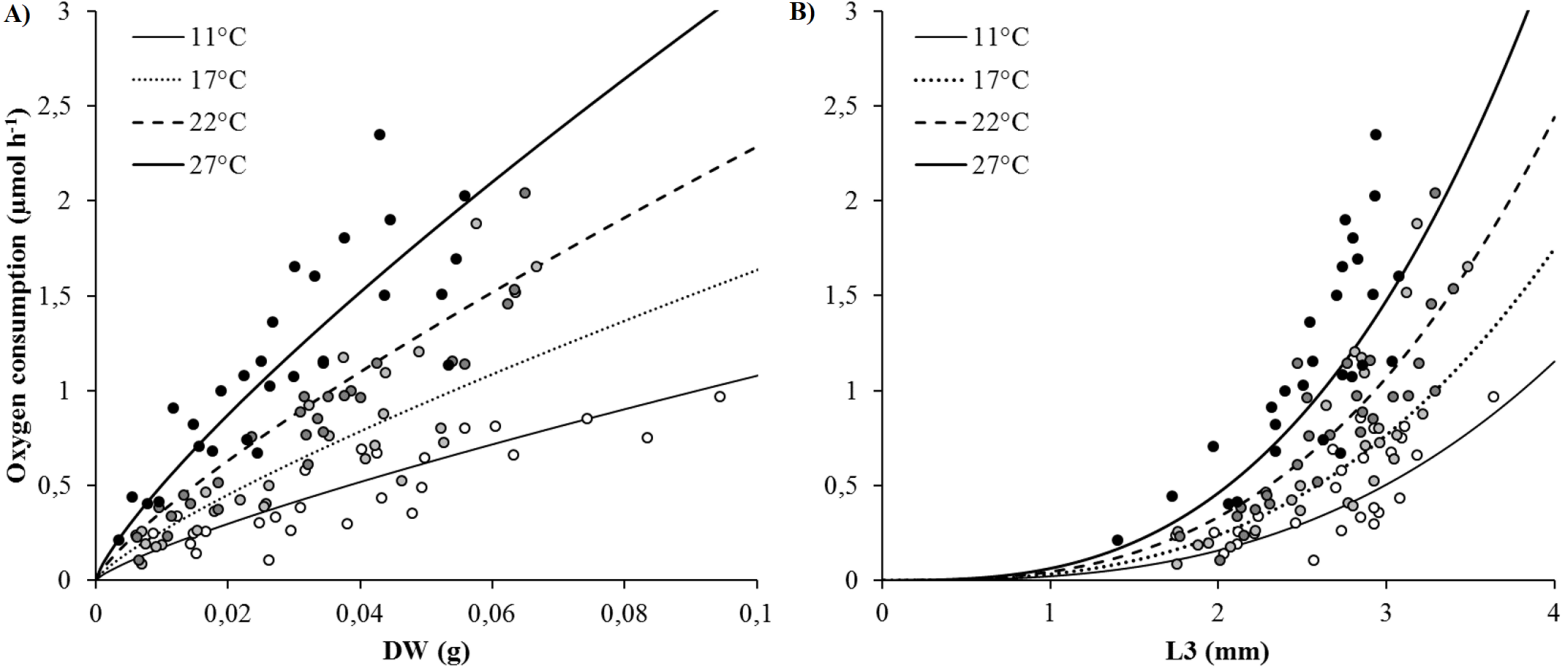
Best-fit models of the effects of temperature and body size (DW and L3) on oxygen consumption rates (*VO*_2_) (μmol h^−1^). (A) Model using dry weight as body size. (B) Model using L3 length as body size. Curves were built from model (parametrization from *T*_*A*_, *k*_1_, *b*: see Table 1) and dots represented raw data (white: 11, light grey: 17, dark grey: 22 and black: 27 °C).

**Figure 3:**
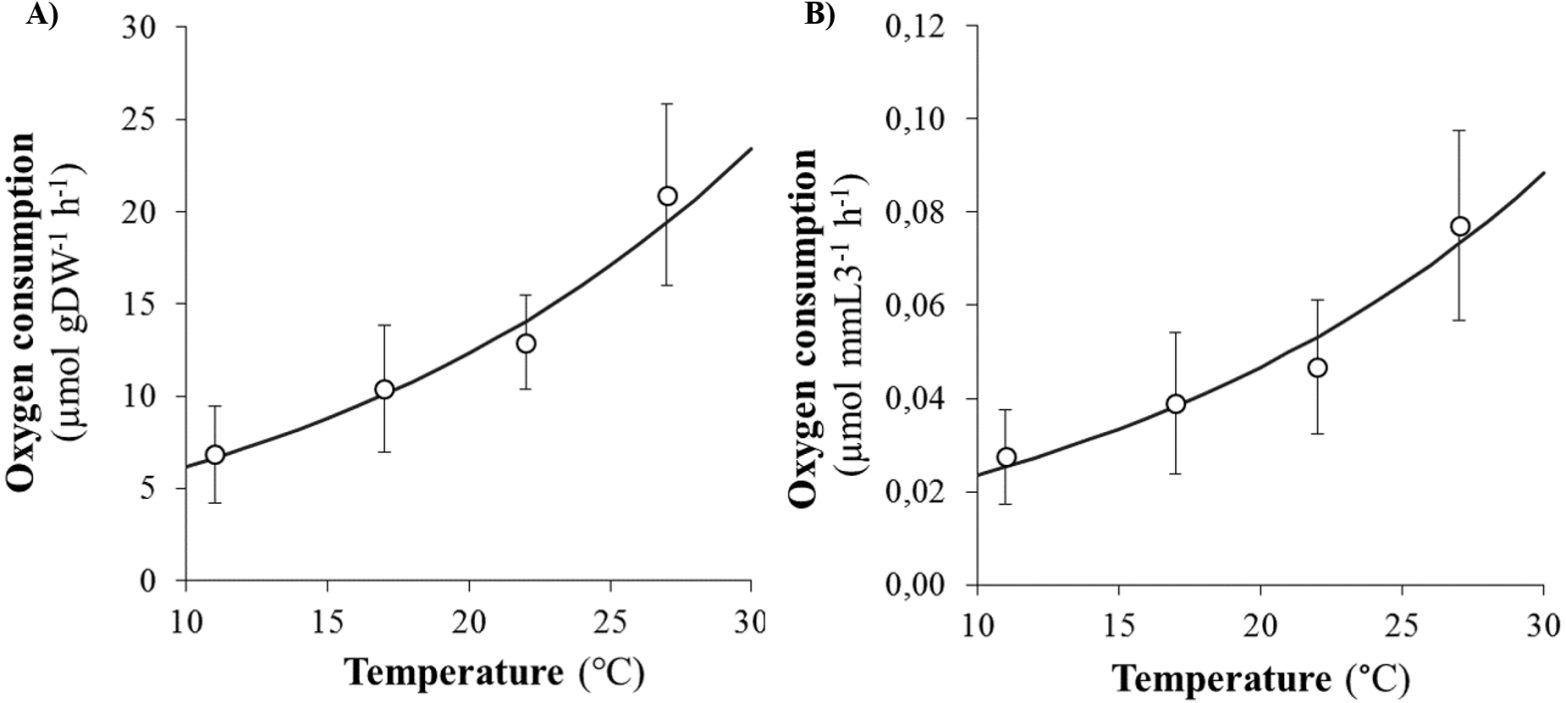
Evolution of oxygen consumption rates of *Hediste diversicolor* according to temperature. Evolution of oxygen consumption rates of *Hediste diversicolor* per unit of body weight (A) or length (B) (µmol gDW^−1^ h^−1^ and μmol mmL3^−1^ h^−1^) (mean ± SD) according to experimental temperatures (11, 17, 22 and 27 °C) estimated from raw data. Data conversion was made using *b* allometric coefficient ** of** 0.80 for DW and 2.83 for L3 and curves were built from model (parametrization from *T*_*A*_, *k*_1_, *b*: see Table 1).

SMR (as proxied by oxygen consumption rate) varied from 0.09 to 2.35 µmol h^−1^ and significantly increased with body size (DW and L3) and temperature (Figs. 2A and 2B). Arrhenius temperature (*T*_*A*_), reaction rate ${\dot {k}}_{1}$ (µmol body size unit^−1^ at *T*_1_: 20 °C) and allometric coefficients (*b*) were estimated for each biometric parameter (WW, DW, L3, TL and NSEG) (Table 1). Results of the best-fit models (DW and L3) are shown in Fig. 2. SMRs by body size unit increased significantly according to temperature whatever the biometric parameter used for standardization (Fig. 3). *Q*_10_ was 2.00 from 10 to 20 °C (estimated from *T*_*A*_) and 2.01 from 17 to 27 °C (estimated from mean ratio 27 vs. 17 °C) when SMR was expressed per DW^−1^.

**Table 1:** Metabolic model parameters for *Hediste diversicolor*. Metabolic model parameters for oxygen consumption (*VO*_2_): means ± SD of the Arrhenius temperature (*T*_*A*_), reaction rate *k*_1_ (oxygen consumption standardized per body size unit at the reference temperature, *T*_1_ = 20 °C, 293.15 K), allometric coefficient (*b*) and *R*^2^ determination coefficient estimated according to different biometric parameters (DW, dry weight; WW, wet weight; L3, length of three first segments; TL, total length; NSEG, number of segments).

Body size	*T*_*A*_ (°K)		*k*_1_ (μmol Body size^−1^ h^−1^)		*b*		*R*^2^
	Mean	SD	Mean	SD	Mean	SD	
DW (g)	5,707.4	425.8	12.33	2.26	0.80	0.06	0.80
WW (g)	5,567.4	448.8	2.60	0.21	0.78	0.06	0.78
L3 (mm)	5,664.0	546.8	0.0467	0.0133	2.83	0.26	0.71
TL (mm)	5,141.9	550.1	0.0050	0.0027	1.30	0.13	0.66
NSEG	5,396.7	730.8	0.0074	0.0059	1.16	0.20	0.46

### Comparison with literature data and determination of active metabolic rate (AMR)

**Figure 4:**
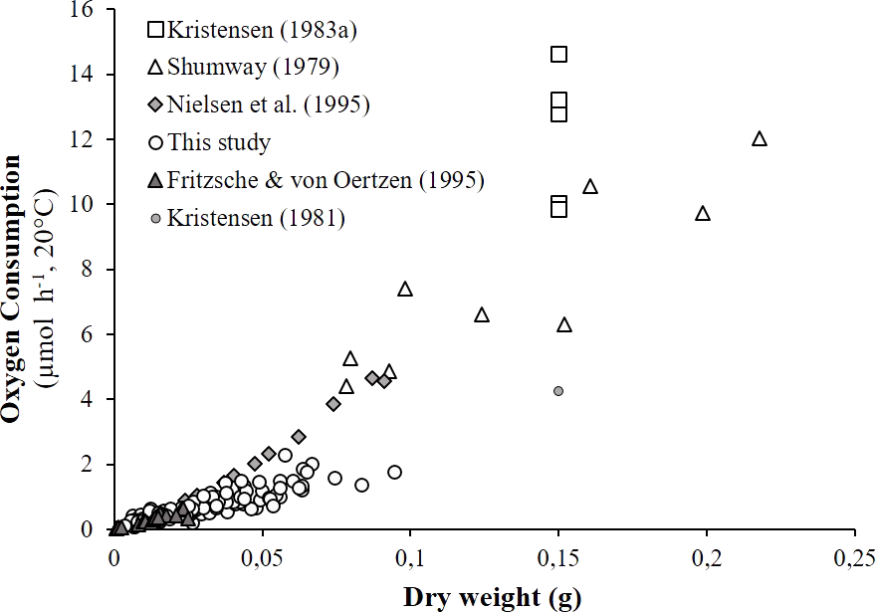
Literature comparison of *Hediste diversicolor* oxygen consumption rates. Oxygen consumption rates of *Hediste diversicolor* (μmol h^−1^) depending on individual body weight (DW in g). Raw data issued from literature and this study were adjusted to reference temperature of 20 °C.

**Figure 5:**
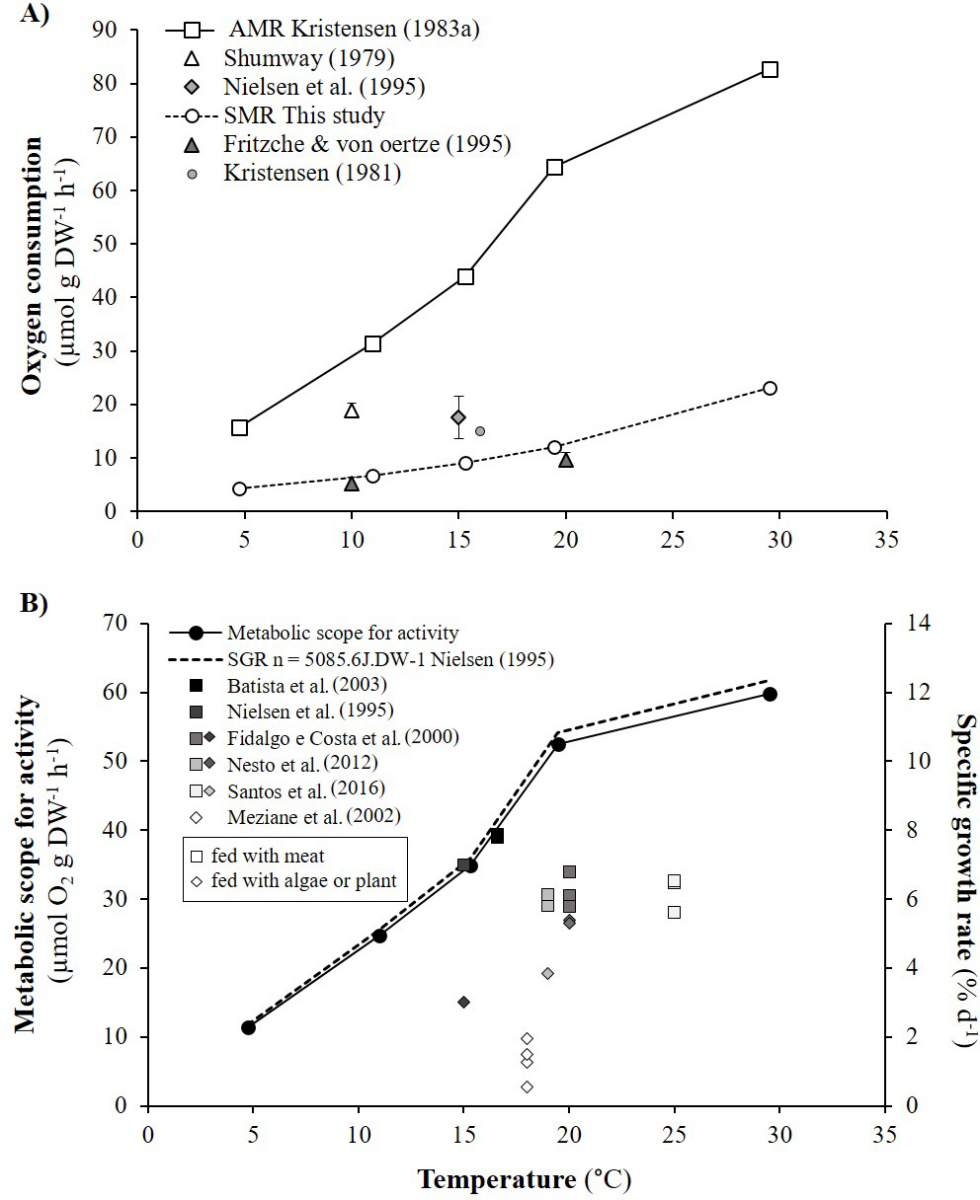
Active metabolic rate (AMR), standard metabolic rate (SMR), metabolic scope for activity (MSA) and specific growth rate (SGR) of *Hediste diversicolor*. (A) Mean oxygen consumption rates of *Hediste diversicolor* per unit of body weight (μmol gDW^−1^ h^−1^) (±SD), for each of experimental temperature. Raw data issued from literature and this study were converted per unit of body weight using allometric coefficient *b* of** 0.80. Data of this study were adjusted to experimental temperature of Kristensen (1983a) and Kristensen (1983b). Maximal and minimal oxygen consumption rate were assumed to be as the active metabolic rate (AMR) and standard metabolic rate (SMR). (B) Metabolic scope for activity (MSA) (μmol O_2_ gDW^−1^ h^−1^) (left) and specific growth rate (SGR) (% d^−1^) (right) calculated according to temperature range (4.7–29.5 °C). Specific growth rate were determined from MSA, converted in J gDW^−1^ d^−1^ using 1 mol O_2_ = 437.67 kJ, day of 24 h, and reported to energy cost for growth (*n*) determined from Nielsen et al. (1995) (*n* = 5086.5 J gDW^−1^). Free dots are SGR of *Hediste diversicolor* from literature. Squares and losanges represented growth rates for worms fed with carnivore (mainly aquaculture food) or herbivorous regime (microalgae or macrophyte).

As detailed in ‘Comparison with literature data and determination of active metabolic rate’, literature values for the oxygen consumption of *H. diversicolor* were converted into µmol h^−1^ and standardized at 20 °C for comparison with the data from this study (Table 2, Fig. 4) or standardized for an individual of 1 gDW for comparison over a large temperature range (Figs. 5A and 5B). In both figures, metabolic rates recorded by Kristensen (1983a), Shumway (1979) and Nielsen et al. (1995) were higher than the oxygen consumption measurements of Fritzsche & Von Oertzen (1995) or in this study. Data from this study (4.3–23.0 µmol gDW^−1^ h^−1^) were qualified as standard metabolic rates (SMR) while maximum oxygen consumption measurements by Kristensen (1983a) (15.7–82.8 µmol gDW^−1^ h^−1^) were defined as active metabolic rates (AMR). AMR:SMR ratio increased up to 20 °C (3.4–5) and decreased thereafter (3.4) (Fig. 5A).

**Table 2:** Literature review of oxygen consumption rates for *Hediste diversicolor*. Review of oxygen consumption rates for *Hediste diversicolor* and metadata about worm body size, temperature, feeding during acclimation, before O_2_ measurements, used device for O_2_ consumption measurements and light exposure. Original Data were extracted from publication using the freeware PlotReader (Bruggeman, 2010) . Body size were converted in DW using DW = 0.15 × WW (this study), DW = 1.28 × AFDW (Wetzel, Leuchs & Koop, 2005).

**Reference**	**Body size (gDW)**	**Temperature (°C)**	**Salinity (psu)**	**Feeding during acclimatation**	**Feeding before O_2_ measurements**	**Contain of chambers**	**Light exposure**	**Original data of Oxygen consumption (different units)**	
	**Min-Max**							**Min-Max**	**Unit**
Shumway (1979)	0.080–0.220	10	No specified	No specified	No specified	Water, without tube	Blackened chambers	0.05–0.14	ml O_2_ h^−1^
Kristensen (1981)	0.0025–0.075	16	20	Sediment, no artificial feeding	Unfed for 48 h	Water, without tube	No specified	104	μg O_2_ g^−1^ h^−1^
		16	20			Water, polyethylene tube	No specified	578	μg O_2_ g^−1^ h^−1^
Kristensen (1983a)	0.068	5	19	Sediment, no artificial feeding	Unfed for 48 h	Water, polyethylene tube	No specified	110,27	μg O_2_ g WW^−1^ h^−1^
		11	19					220,59	μg O_2_ g WW^−1^ h^−1^
		15	19					308,18	μg O_2_ g WW^−1^ h^−1^
		19	19					452,41	μg O_2_ g WW^−1^ h^−1^
		30	19					580,69	μg O_2_ g WW^−1^ h^−1^
Fritzsche & Von Oertzen (1995)	0.0006–0.025	10	5	Sediment, no artificial feeding	Unfed for 12–24 h	Water, ashed sediment	Darkness	3.5–11.6	mJ h^−1^ mg AFDW^−1^
		20	5					10.6–21.1	mJ h^−1^ mg AFDW^−1^
Nielsen et al. (1995)	0.024–0.091	15	15	Glass tubes, shrimp meat	Unfed during 10–14 days	Water, glass tubes	No specified	13.99–72.97	μl O_2_ h^−1^
This study	0.003–0.094	11	18	Water, seabass feces	Unfed for 24 h	Water, without tube	Dark	6,85	μmol O_2_ gDW^−1^ h^−1^
		17	18					10,41	μmol O_2_ gDW^−1^ h^−1^
		22	18					12,91	μmol O_2_ gDW^−1^ h^−1^
		27	18					20,90	μmol O_2_ gDW^−1^ h^−1^

### Metabolic scope for activity and maximum growth rates

Metabolic scope for activity (MSA) was determined from AMR and SMR over the temperature range studied by Kristensen (1983a) (Fig. 5A). MSA varied from 11.4 to 59.8 µmol gDW^−1^ h^−1^, i.e., 120.1 to 627.6 J gDW^−1^ d^−1^. Maximum growth rates (*μ*_max_) were determined from MSA and energy cost for growth (*n*). The *μ*_max_ significantly increased with temperature, specifically from 5 to 20 °C with a lower slope between 20 and 30 °C (Fig. 5B). With *n* equivalent to 5086.5 J gDW^−1^ (Nielsen et al., 1995), *μ*_max_ varied from 2.4 to 12.3% d^−1^ according to temperature. Published specific growth rates (SGR) vary from 0.5 to 7.9% d^−1^ when *H. diversicolor* were maintained between 15 and 25 °C (Fig. 5B). Growth rates were higher for worms fed (5.6–7.9% d^−1^) with meat rather than algae or plant material (0.6–5.3% d^−1^) (Fig. 5B). The growth curve estimated with *n* from the literature fitted the highest growth rate (7.9% d^−1^) recorded with worms fed with seabream dry food at 16.6 °C (Batista et al., 2003).

## Discussion

The objective of this study was to gain insight into *Hediste diversicolor* metabolic rates and metabolic scope for activity across broad ranges of body size and temperature. Firstly, relationships between various weight and length metrics were determined. Secondly, experiments were conducted to test the simultaneous effects of body size and temperature on *H. diversicolor* standard metabolic rate (i.e., oxygen consumption rate) to identify critical parameters (allometric coefficient, reaction rate at 20 °C, Arrhenius temperature) and build an empirical model for this species. Finally, based on comparison with published data, metabolic scope for activity and maximum specific growth rates were estimated across a large temperature range.

### Biometrics of *Hediste diversicolor*

Biometric measurements on *H. diversicolor* revealed key relationships between various proxies for weight (WW, DW) and length (L3 and TL) (Fig. 1). From weight or total length of *H. diversicolor*, it is easy to estimate dry weight, length of three first segments (L3) and total number of segments. Indeed, DW corresponded to 15% of WW, in agreement with the findings of Wetzel, Leuchs & Koop (2005). Note that experiment was done at 18 psu. At other salinities, this relationship may differ since the water content of an individual decreases with increasing water salinity (Fletcher, 1974). Thus, Kristensen (1984) and Durou, Mouneyrac & Amiard-Triquet (2008) estimated water content to vary from 84 to 87%. Length of three first segments (L3) was 2.5% of TL, considering a minimum of 1.44 mm for L3. These relationships have not been published before. According to Dales (1950) and Scaps (2002), the relationship between TL and NSEG can change during the two growth phases in polychaetes, i.e., the segmental (initial life stages) and weight growth (juvenile and adult) phases. Therefore, worms with the same number of segments may not have the same age or body size. Nevertheless, using this study, the number of segments may be estimated from total length, considering mean segment length of 0.85 mm for worms ranging for 15 and 85 mm. Finally, from L3 or TL, it is easy to estimate the weight of worms using WW (mg) = 9.85 × TL (cm)^1.92^ and WW (mg) = 5.33 × L3 (mm)^3.69^ and DW (mg) = 0.15 × WW (mg). From the established relationships, a worm of 10 cm-commercialized-size (Scaps, 2002) measures 3.94 mm L3, and weighs 819.29 mg WW and 122.89 mgDW. The established size-weight relationship was similar to those observed by Durou, Mouneyrac & Amiard-Triquet (2008) when length was characterized by TL, but was significantly different when length was defined by L3 (Fig. 1) (Gillet, 1990; Durou, Mouneyrac & Amiard-Triquet, 2008). Differences may be attributed to formalin conservation: when nereid worms are placed in 4% formalin the body muscles contract and the animals die in this state (Kristensen, 1984). Total worm length was 1.27-fold lower after formalin treatment (Kristensen, 1984). There is no information about effect of formalin on L3 in the literature but our results suggest that L3 might be significantly reduced by muscle contraction. To avoid this kind of bias, length determination should be carried out on live worms using pictures, as done in this study.

Among all the biometric measurements tested, DW and L3 were the most accurate descriptors of *H. diversicolor* body size. Indeed, in contrast to wet weight, dry weight did not depend on salinity or osmoregulation processes. In contrast to NSEG and TL, L3 is not affected by stress. In stressful conditions, worms are frequently damaged at their extremities due to physical trauma and autotomy so number of segments and total length are less representative measurements (Gillet, 1990; Durou, Mouneyrac & Amiard-Triquet, 2008). Finally, L3 is probably the best proxy of body size since length, unlike weight, does not fluctuate according to reserve or reproductive status (Kooijman, 2010). Thus, analyses of body size effects on metabolism should be based on these two parameters (DW and L3).

### Effect of body size and temperature on standard metabolic rate (SMR)

In many physiological activities, absolute body size is the most important factor determining the rate of processes (Bertalanffy, 1957). Oxygen consumption rates of *Hediste diversicolor* increase with body size as has already been observed (Shumway, 1979; Nielsen et al., 1995), whatever biometric parameters are used. In several studies, oxygen consumption rate is expressed in biomass units without applying allometric rules (Kristensen, 1983a; Fritzsche & Von Oertzen, 1995). For *H. diversicolor*, the *b* allometric coefficient was 1.2 for individuals weighing between 30 and 90 mgDW (Nielsen et al., 1995) and 0.69 for bigger ones (80 and 220 mgDW) (Shumway, 1979). The *b* allometric coefficient is typically higher in younger individuals (up to 1) than older ones (down to $ \frac{2}{3} $; Riisgård, 1998). The *b* coefficient determined in this study was intermediate (0.8) for worms of the same range of body weight as in Nielsen et al. (1995), suggesting a difference in physiological status. In this study, allometric coefficients (*b*) were also determined for wet weight and length parameters: 0.78 (WW), 0.8 (DW) and 2.83 (L3) for individuals ranging from 4 to 94 mgDW (15 to 85 mmTL). Results are consistent with commonly accepted allometric coefficients, i.e., (*b*) ranging from 0.67 to 1 (weight) and from 2 to 3 (length), depending on life stage and environmental conditions (Bertalanffy, 1957). TL and NSEG were probably underestimated (as discussed in ‘Biometrics of *Hediste diversicolor*’), making *b* lower than the theoretical range for length (2–3 vs. *b*_TL_ = 1.3 and *b*_NSEG_ = 1.16). The determined allometric coefficients will be very useful for other studies when it comes to converting oxygen consumption according to body size.

Simultaneously, an empirical model constructed from the oxygen consumption (*VO*_2_) database permits to determine Arrhenius temperature (*T*_*A*_) and reaction rates (${\dot {k}}_{1}$) at the reference temperature (20 °C, 293.15 K) for each biometric parameter. Mean oxygen consumption rates of 12.33 µmol gDW^−1^ h^−1^ and 0.0467 µmol mmL3^−1^ h^−1^ at 20 °C were estimated (Figs. 2A and 2B). The Arrhenius temperature (*T*_*A*_) was above 5,700 K for DW and L3. The higher the Arrhenius temperature, the less activation energy is required, indicating that chemical reactions and metabolism are favored in higher temperatures. The *T*_*A*_ found for *H. diversicolor* was consistent with the *T*_*A*_ found for other intertidal invertebrates, such as several bivalve species, i.e., *T*_*A*_ around 5,800 K (Van der Veer, Cardoso & Van der Meer, 2006). When studying the activities and thermal sensitivities of key enzymes of aerobic metabolism in the polychaete *Arenicola marina,* Sommer & Pörtner (2002) found that *T*_*A*_ ranged from 3,368 to 6,677 K. An Arrhenius temperature of 5,700 K corresponds to a Van’t Hoff coefficient *Q*_10_ of 1.9 between 10 and 20 ° C, which is consistent with values for *H. diversicolor* published by Kristensen (1983a) (*Q*_10_ = 2.2 for 5–16 °C, *Q*_10_ = 1.8 for 16 to 30 °C) and Fritzsche & Von Oertzen (1995) (*Q*_10_ = 1.5 to 2 for 10–20 °C).

### Comparison with the literature and determination of active metabolic rate (AMR)

The effects of body size and temperature on oxygen consumption in *H. diversicolor* have been documented in a few studies (Shumway, 1979; Kristensen, 1983a; Fritzsche & Von Oertzen, 1995; Nielsen et al., 1995). Comparisons with the results of this study were possible using the established weight-size relationships and empirical model parameters (Figs. 4 and 5). As expected, the recorded oxygen consumptions of *H. diversicolor* were equivalent to observed rates (Fritzsche & Von Oertzen, 1995) and were defined as standard metabolic rates (SMR). SMRs were 2- to 5-fold lower than those reported by other authors (Shumway, 1979; Kristensen, 1983a; Nielsen et al., 1995). Differences may be related to the fact that worms in artificial tubes are particularly active (Kristensen, 1981; Kristensen, 1983b). Indeed, ventilation amplitude and duration of worms contained in artificial tubes were shown to be 2- and 3-fold higher than in natural sediment for two species of Nereidae (Kristensen, 1983b). Authors argued that this difference was due not only to higher resistance in natural sediment but also to organism stress. Polychaetes alternated between resting periods and bursts of ventilator activity. Oxygen uptake of three species of *Nereis* increased during active ventilation periods observed in polyethylene tubes, in contrast to during ventilation pauses when worms were removed from the tubes and put in a closed respirometer (Kristensen, 1981). Similar conditions in this study explained low oxygen consumption measurements: the *H. diversicolor* individuals were at rest and did not show significant movement. The large difference in oxygen consumption rate from inactivity to active ventilation was not simply due to the animal’s mechanical work to generate a current but also because ventilation in nereids is accompanied by peristaltic movements of practically every muscle in the body. This internal work—to circulate body fluids and activate the gut—accounts for a substantial fraction of the energy (Kristensen, 1981). For these reasons, oxygen consumption rates in Kristensen (1983a) were used to specify active metabolic rates (AMR).

### Metabolic scope for activity and specific growth rate

Metabolic scope for activity (MSA) was determined by difference between active metabolic rate (AMR) and standard metabolic rate (SMR) (Norin & Malte, 2011). MSA, which represents energy allocated for activity, growth and reproduction, ranged from 120.1 to 627.6 J gDW^−1^ d^−1^ at different temperatures. SMR and AMR both increased with temperature but not linearly and not in proportion to one another. This is due to lower availability of oxygen and high temperature for active individuals. The ratio between AMR and SMR increased with temperature, from 3.4 to 5 between 10 to 20 °C, and thereafter decreased towards 3.4 at 30 °C. *H. diversicolor* oxygen uptake and ventilation amplitude are known to increase with temperature (Kristensen, 1983a) with higher *Q*_10_ from 5.5 to 16 °C (2.91) than from 16 to 30 °C (1.01). From the analyses of ventilation requirement (oxygen consumed/water ventilated), optimum temperatures for *H. diversicolor* were seen between 5 to 16 °C (Kristensen, 1983a). From 16 °C to 20 °C ventilation activity continued to increase with a lower slope but then decreased from 20 to 30 °C (Kristensen, 1983a). This decrease above 20 °C may be due to the lower AMR:SMR ratio and, in consequence, lower metabolic scope for activity.

Beyond some temperature threshold, metabolic scope for activity can be reduced due to high maintenance costs, temperature stress and lower oxygen availability. In this study, this threshold was not reached. Nevertheless, a plateau was observed for MSA between 20 and 30 °C and for oxygen consumption rates between 22 °C and 27 °C. At temperatures above 30–35 °C, ventilation of *H. diversicolor* is known to decrease and eventually cease (Kristensen, 1983a). Thus, at 35 °C the metabolic scope for activity would probably be low or null. Further experiments would be needed to test this, notably in a broader perspective such as the effect of global warming on metabolic scope for activity of *H. diversicolor*.

From the established curve of metabolic scope for activity and the energy cost for growth determined by (Nielsen et al., 1995), this study highlighted an increase in maximum specific growth rate (*μ*_max_, 2.4–12.3%) across a large temperature range. The *μ*_max_ temperature curve fits well with published maximum specific growth rates (SGR) for worms fed with shrimp at 15 °C (7% d^−1^) (Nielsen et al., 1995) or with dry food (for seabream or for ornamental fish) between 13.8 and 16.6 °C (7.8% d^−1^) (Batista et al., 2003). Mid-range SGRs were observed between 19 and 25 °C (5.6–6.8% d^−1^) for worms fed with protein-rich fish or larval food (Aquagold, Moist Sole, Tetramin, Lansy, Larviva-Biomar) (Fidalgo e Costa, Narciso & Cancela da Fonseca, 2000; Nesto et al., 2012; Santos et al., 2016). The lowest SGRs (0.5–5.4%) were recorded for worms fed with microalgae (Nielsen et al., 1995), macrophyte detritus (spartina, Halimione, Salicornia, enteromorpha, microalgae) (Meziane & Retiere, 2002) or soy and pollen (Fidalgo e Costa, Narciso & Cancela da Fonseca, 2000). *H. diversicolor* may shift from predatory/surface deposit-feeding to suspension feeding according to the concentration of phytoplankton (Nielsen et al., 1995). Higher growth rates observed with meat vs. algae may be partly related to the extra costs of pumping (filter feeding) and differential assimilation efficiency (Nielsen et al., 1995). In the same way, plant detritus consisted of refractory rather than labile matter, contained less protein and fat, and was less easily assimilated. On a “plant”-based diet, available and/or assimilated energy were not high enough to meet potential AMR.

Metabolic scope for activity (MSA) provides the margin of energy available for any physiological work. The value of 5,100 J gDW^−1^ we estimated from the work of Nielsen et al. (1995) is close to the average cost of 7,200 J gDW^−1^ estimated for many different types of organism (Wieser, 1994). This value fits with the highest growth rates of the literature. MSA has been proposed as an indicator of animal welfare and physiological conditions in aquaculture (Claireaux & Lefrançois, 2007). Even if some uncertainties remain in the observed growth rates or estimation of cost for growth, the medium SGRs recorded in the literature (with *μ*_max_ and indeed MSA used as reference) probably indicate that optimal culture conditions were not met in these experiments (Fidalgo e Costa, Narciso & Cancela da Fonseca, 2000; Nesto et al., 2012; Santos et al., 2016). Factors to investigate to explain these results include false *ad libitum* food conditions, oxygen availability, the redox state of the sediment, density effects and other stresses.

## Conclusions and Perspectives

This study highlights key relationships and parameters to improve understanding of the morphology, metabolic rate, metabolic scope for activity and maximum specific growth rate of *H. diversicolor*, based on published data and the results of new experiments. This study provides useful data for bioenergetic models to predict metabolic rates and growth in different temperature conditions. Scenarios combing different temperatures, densities and body sizes could be tested to identify ideal aquaculture conditions for *H. diversicolor*. These results could have interesting applications in the fields of ecology and biogeochemistry in the context of global warming, especially considering the fundamental role of *H. diversicolor* in coastal ecosystems. Nevertheless, further studies will be necessary to highlight the contributions of worm metabolism and their influence on oxygen demand at the water-sediment interface via bioirrigation and bioturbation activities.

##  Supplemental Information

10.7717/peerj.5675/supp-1 Supplemental Information 1 Raw Data Oxygen consumption and biometric measurements.

## Additional Information and Declarations

### Data Availability

The following information was supplied regarding data availability: The dataset is provided in Supplemental File.
